# Enhanced charge density wave with mobile superconducting vortices in La_1.885_Sr_0.115_CuO_4_

**DOI:** 10.1038/s41467-023-36203-x

**Published:** 2023-02-09

**Authors:** J.-J. Wen, W. He, H. Jang, H. Nojiri, S. Matsuzawa, S. Song, M. Chollet, D. Zhu, Y.-J. Liu, M. Fujita, J. M. Jiang, C. R. Rotundu, C.-C. Kao, H.-C. Jiang, J.-S. Lee, Y. S. Lee

**Affiliations:** 1 Stanford Institute for Materials and Energy Sciences, SLAC National Accelerator Laboratory, 2575 Sand Hill Road, Menlo Park, CA 94025 USA; 2 Department of Materials Science and Engineering, Stanford University, Stanford, CA 94305 USA; 3 Stanford Synchrotron Radiation Lightsource, SLAC National Accelerator Laboratory, Menlo Park, CA 94025 USA; 4 PAL-XFEL, Pohang Accelerator Laboratory, Gyeongbuk, 37673 South Korea; 5 Institute for Materials Research, Tohoku University, Katahira 2-1-1, Sendai, 980-8577 Japan; 6 Linac Coherent Light Source, SLAC National Accelerator Laboratory, Menlo Park, CA 94025 USA; 7 Department of Applied Physics, Stanford University, Stanford, CA 94305 USA; 8 SLAC National Accelerator Laboratory, Menlo Park, CA 94025 USA

**Keywords:** Superconducting properties and materials, Electronic properties and materials

## Abstract

Superconductivity in the cuprates is found to be intertwined with charge and spin density waves. Determining the interactions between the different types of order is crucial for understanding these important materials. Here, we elucidate the role of the charge density wave (CDW) in the prototypical cuprate La_1.885_Sr_0.115_CuO_4_, by studying the effects of large magnetic fields (*H*) up to 24 Tesla. At low temperatures (*T*), the observed CDW peaks reveal two distinct regions in the material: a majority phase with short-range CDW coexisting with superconductivity, and a minority phase with longer-range CDW coexisting with static spin density wave (SDW). With increasing magnetic field, the CDW first grows smoothly in a manner similar to the SDW. However, at high fields we discover a sudden increase in the CDW amplitude upon entering the vortex-liquid state. Our results signify strong coupling of the CDW to mobile superconducting vortices and link enhanced CDW amplitude with local superconducting pairing across the *H* − *T* phase diagram.

## Abstract (web summary)

Superconductivity in the cuprates is known to be intertwined with charge and spin density waves. Here, the authors study the prototypical cuprate La_1.885_Sr_0.115_CuO_4_ via x-ray scattering and discover a sudden increase in the charge-density-wave amplitude upon entering the superconducting-vortex-liquid state at high magnetic field.

## Introduction

High-*T*_*c*_ cuprates are the prominent example of a strongly correlated electronic system, featuring a rich phase diagram marked by novel types of order^1^. Tremendous effort has been devoted to studying these orders to understand the unconventional normal state and high-*T*_*c*_ superconductivity^2^. It is recognized that such complex systems are susceptible to electronic inhomogeneity^3^, which may arise intrinsically due to electronic interactions^4^ or for extrinsic reasons, such as chemical disorder^5^. Indeed, nanoscale spatial variations in electronic properties of the cuprates have been observed and may be relevant to their physics^6,7^. This issue is manifest in underdoped La_2_CuO_4_-based cuprates such as La_2−*x*_Sr_*x*_CuO_4_ (LSCO), where long-range SDW (with correlation length of hundreds of unit cells) coincides with bulk superconductivity^8^. Consensus has yet to be reached regarding whether these orders coexist uniformly, or exist within distinct regions. Both scenarios have been suggested in theoretical studies^9–11^, while it has been difficult to determine experimentally^12–14^.

Recent observations of CDW order across cuprate families shed new perspective on the interplay between density wave orders and superconductivity^1,2,8^. When SDW order is absent, such as in Y-based cuprates (YBCO) around 1/8 doping, clear competition between CDW and superconductivity has been observed^15,16^. When both SDW and CDW are present in the La_2_CuO_4_-based cuprates, they appear to form a spin-charge stripe order pattern, indicated by nearly commensurate wave vectors (*q*_cdw_ ~ 2*q*_sdw_)^8^. There are theoretical and experimental evidences of mutual cooperation between CDW and SDW order parameters when such commensuration is satisfied^17,18^. Here the interaction between density waves and superconductivity appears more than simple competition. For example, the SDW onset temperature (*T*_sdw_ as measured by neutron scattering) in LSCO around 1/8 doping is similar to the superconducting *T*_*c*_^8^, and a putative two-dimensional superconductivity called pair density wave has been associated with the stripe order^2,19^. To elucidate the intrinsic behavior of these orders and distill universal features among different cuprates, it is important to carefully interpret observations in light of the inhomogeneity.

X-ray scattering combined with high magnetic fields provides a unique window into the nature of the coexistence of CDW, SDW, and superconductivity. If there is inhomogeneity on length scales larger than the CDW correlation length (typically tens of unit cells^15,16,18,20–22^), it should be manifest in the evolution of the CDW peaks when the strength of the various orders is tuned by *T* and *H*. We investigate La_1.885_Sr_0.115_CuO_4_ where the stripe order is robust and of comparable strength to superconductivity (*T*_sdw_ ~ *T*_*c*_)^23^. Our state-of-the-art x-ray free electron laser measurements (see Methods for details) reveal evidence for two types of CDW orders in the same sample, distinguished by different correlation lengths and distinct *T*- and *H*- dependences. Most importantly, we further uncover a strong connection between the short-range CDW correlations and mobile superconducting vortices, which bears important implications regarding the nature of the superconducting transition in the cuprates.

## Results

**Fig. 1:**
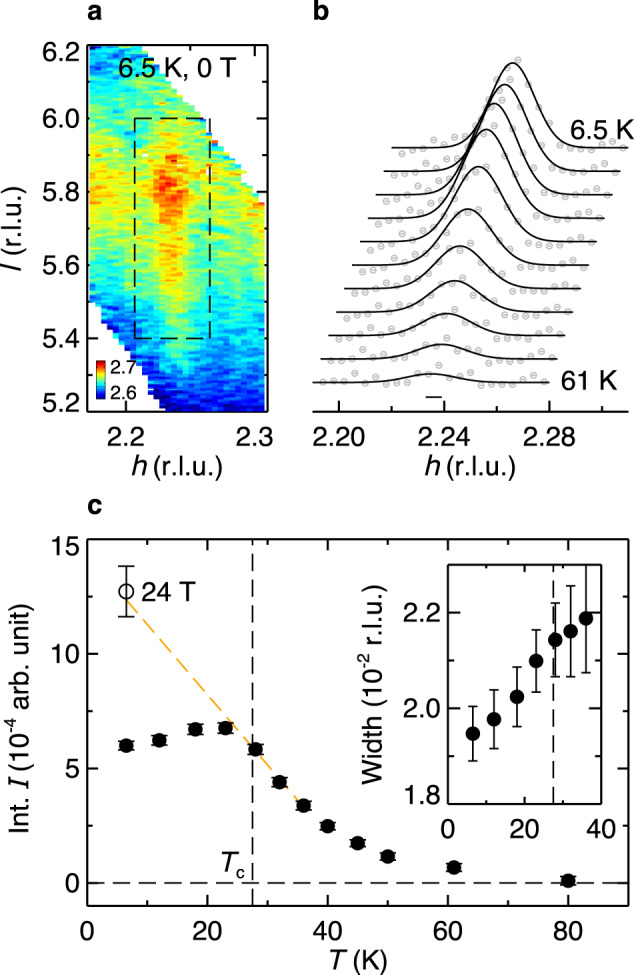
Temperature dependence of CDW in LSCO. **a** CDW intensity map measured at 6.5 K, 0 T, projected onto *hl* plane, integrated over *k* [−0.02,0.02] r.l.u. The dashed black rectangle encircles the CDW peak. **b** Temperature-dependent *h*-cuts through the CDW peak. Solid lines are single-peak fits to the data. A linear background has been subtracted, and data are shifted for clarity. The horizontal bar represents instrumental resolution. **c** Integrated CDW intensities extracted from the single-peak fits. The inset shows the corresponding peak width. The orange dashed line is a linear extrapolation of the CDW intensities at 28 K, 32 K, and 36 K to lower temperatures *T* < *T*_*c*_, as described in the main text. The open circle shows the CDW intensity measured at 6.5 K, 24 T. Error bars represent one standard deviation.

We first address the issue of inhomogeneity by examining the CDW *T*-dependence in zero magnetic field. As shown in Fig. 1a, the CDW peak appears as a rod of intensities along the *l* direction, which demonstrates its quasi-two-dimensional nature^15,16,20,22^ (more in Supplementary Figs. 5, 6). To focus on the CDW correlations within the CuO_2_ planes, the CDW intensities are integrated and projected along *h*. As a first analysis, a single Gaussian peak is found to fit the data well (Fig. 1b). Consistent with previous hard x-ray measurements^20,22^, the CDW intensities become appreciable below ~80 K (Fig. 1c). The onset with upward concavity is a common feature of the CDW in the cuprates, which indicates a lack of a long-range CDW transition^24^. While our hard x-ray scattering measurements are sensitive to the quasi-static lattice distortions associated with the CDW, other resonant x-ray scattering studies have detected CDW in LSCO up to higher temperatures^18,21^. This may be related to dynamical CDW fluctuations which persist over a much larger temperature range and can have important implications for high-*T*_*c*_ cuprates^25–28^.

The CDW intensity reaches a maximum near the superconducting *T*_*c*_ before getting suppressed at lower temperatures, indicating competition between CDW and superconductivity. However, unlike a homogeneously weakened order, the CDW peak width keeps decreasing for *T* < *T*_*c*_ (Fig. 1c inset), which implies a growing CDW correlation length. Such contradictory behavior between the CDW intensity and correlation length has been observed, though with varying clarity, in previous x-ray measurements for LSCO at similar doping levels^20–22^ (also see Supplementary Fig. 7), and is in stark contrast with YBCO, where both the CDW intensity and correlation length decrease for *T* < *T*_*c*_^15,16^. This indicates that mere competition between CDW and superconductivity is insufficient to explain the CDW behavior in LSCO.

**Fig. 2:**
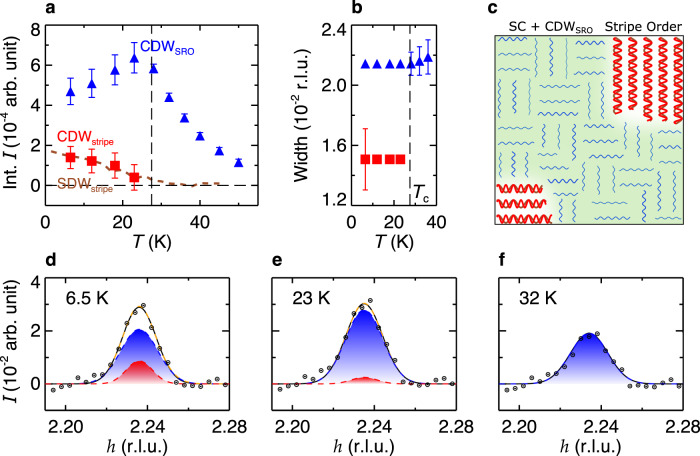
Two-component decomposition of the CDW order in LSCO. **a** Integrated intensities for CDW_SRO_ and CDW_stripe_ extracted from the two-component fits. The brown dashed line shows the scaled SDW intensities measured by neutron scattering^23^. **b** Corresponding peak width for CDW_SRO_ and CDW_stripe_. The widths are fixed for *T* < *T*_*c*_ for the two-component fits, as described in the main text. **c** A pictorial illustration of the mixed phases in LSCO at low temperatures, which consist of superconducting regions with suppressed CDW_SRO_, and separate regions dominated by spin-charge stripe order. **d**–**f** Representative data and corresponding fits at 6.5 K, 23 K, and 32 K, respectively. Orange, blue, and red dashed lines show the total, CDW_SRO_, and CDW_stripe_ of the two-component fits, respectively. For comparison, the single-peak fits are shown in solid black lines. Blue and red shadings illustrate contributions from CDW_SRO_ and CDW_stripe_, respectively. Error bars represent one standard deviation.

As alluded to above, the cause here is the SDW order which onsets at *T*_sdw_ ~ *T*_*c*_ in LSCO and enhances CDW^18,23^, but does not coexist with CDW in YBCO^29^. To further elucidate the thermal evolution of the CDW, we decompose the CDW peak into two components: CDW_stripe_ to describe the SDW-enhanced component, and CDW_SRO_ (short-range order) to account for the competition with superconductivity. CDW_SRO_ is expected to behave similarly to the CDW in LSCO near optimal doping (*x* ~ 0.145) where the SDW order is absent^18^. Since the CDW peak width there is weakly *T*-dependent at low temperatures^18^, we fix the CDW_SRO_ peak width for *T* < *T*_*c*_ to that extracted at *T*_*c*_ in the single-peak fit, where the SDW order is just about to develop (Fig. 2b). This CDW_SRO_ component has correlation length of *ξ*_SRO_ = 2/FWHM = 56(2) Å. As for CDW_stripe_, to a first approximation we also assume a *T*-independent peak width. To extract this width, we fit the *T* = 6.5 K data with the sum of the aforementioned CDW_SRO_ peak and a second peak. The best fit yields a sharper component (CDW_stripe_) with correlation length *ξ*_stripe_ = 80(11) Å. Interestingly, *ξ*_SRO_ is comparable to that in YBCO at low temperatures^15,30^, while *ξ*_stripe_ is comparable to that in the canonical stripe ordered cuprates La_1.48_Nd_0.4_Sr_0.12_CuO_4_ and La_2−*x*_Ba_*x*_CuO_4_ (LBCO) for which the CDW correlation length is ~1/4 of the SDW correlation length^31,32^ (here, the SDW correlation length in LSCO is ~300 Å^33^). Fixing the widths for these two components and allowing the respective intensities to vary, such constrained two-component fitting provides an alternative and better account of the data (Fig. 2d–f, Supplementary Fig. 8). Consistent with our hypothesis, the fitting results show that the CDW *T*-dependence for *T* < *T*_*c*_ can be described in terms of the weakening of CDW_SRO_ and the enhancement of CDW_stripe_ (Fig. 2a). In particular, the CDW_stripe_ intensity follows the SDW intensity measured by neutron scattering^23^ (Fig. 2a), consistent with their cooperative interactions.

Such a decomposition provides a natural explanation of the seemingly contradictory behavior of the intensities and widths in the single-peak analysis. The combined effect of a weakened CDW_SRO_ (broad peak) and an enhanced CDW_stripe_ (sharp peak) results in the net reduction of the integrated intensity simultaneously with a narrowing of the width. In fact, previous local probe measurements (such as *μ*SR^12^ and NMR^34^) have indicated heterogeneous phases, and scattering studies which measure average correlations in the bulk have also provided evidence for charge inhomogeneities^35^. The high statistical quality of the zero-field data and resulting success of the two-component analysis to the CDW *T*-dependence strongly support the assertion that the CDW order is indeed heterogeneous for *T* < *T*_*c*_ in LSCO, with coexisting CDW_SRO_ and CDW_stripe_ components. This further indicates superconductivity and SDW does not coexist in a uniform phase: the CuO_2_ plane segregates into superconducting regions with suppressed CDW_SRO_, and separate regions dominated by spin-charge stripe order (CDW_stripe_ commensurate with SDW), as illustrated in Fig. 2c.

**Fig. 3:**
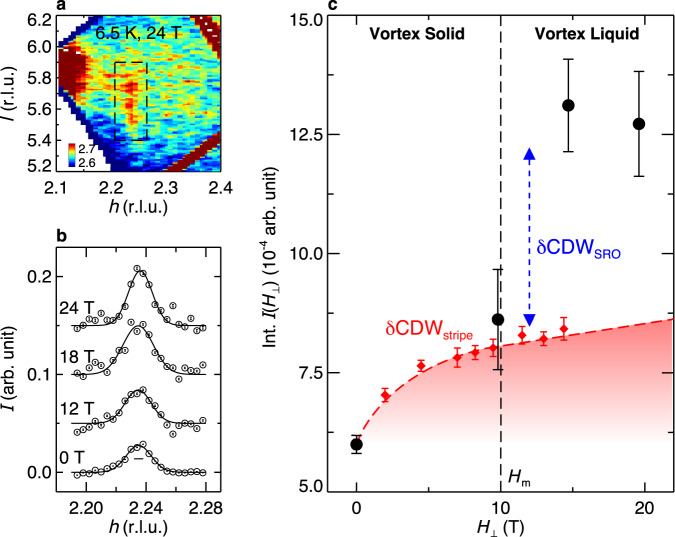
Magnetic-field dependence of CDW in LSCO. **a** CDW intensity map measured at 6.5 K, 24 T, projected onto *hl* plane. **b** Magnetic-field-dependent *h*-cuts through the CDW peak. Solid lines are one-Gaussian fits to the data. A linear background has been subtracted, and data are shifted for clarity. The horizontal bar represents instrumental resolution. **c** Integrated CDW intensities as a function of magnetic field. Note here the magnetic-field projection perpendicular to the CuO_2_ planes *H*_⊥_ is plotted. The vertical dashed line marks the vortex-melting field *H*_m_ for LSCO at *T* ~ 6.5 K determined by in-plane resistivity measurements^41^. The red diamonds are field-dependent SDW intensities measured by neutron for LSCO^14^, scaled such to deduce the magnetic-field-induced enhancement of CDW_stripe_, as described in the main text. The red dashed line and red shading are guide to the eye. Error bars represent one standard deviation.

With this picture in mind, we turn to the evolution of the CDW order under high magnetic field. Fig. 3a shows the intensity collected at 24 T. The persistence of the rod-like scattering shows that the CDW correlations remain two-dimensional (more in Supplementary Fig. 5). We note that we find no evidence of three-dimensional CDW order at integer *l* position (here *l* = 6) (see Supplementary Fig. 13), which has been observed in YBCO at a similar doping level and magnetic field^30^. This difference could be related to the different value for *H*_*c*2_ and/or how the CDW order on neighboring CuO_2_ planes interacts^36^. Future measurements at even higher magnetic fields may provide more insight.

Focusing on effects of field, we plot scans through the CDW peak in Fig. 3b. An increase in the CDW intensity with magnetic field is clearly observed. Although the statistical quality of the high-field data (constrained by magnet recovery time between magnetic pulses, see Methods) hinders a two-component analysis, the successful decomposition at zero field enables us to surmise the respective evolution of CDW_SRO_ and CDW_stripe_ in magnetic field. Assuming the proportionality between CDW_stripe_ and SDW (Fig. 2a) holds in magnetic field, which is supported by previous measurements in LSCO and LBCO^22,37,38^ (see Supplementary Fig. 9 and corresponding discussion), we use the SDW magnetic-field dependence^14^ to infer the CDW_stripe_ enhancement (with no adjustable parameters), *δ*CDW_stripe_(*H*_⊥_) = CDW_stripe_(0) × [SDW(*H*_⊥_)/SDW(0) − 1], where *H*_⊥_ is the applied field projection perpendicular to CuO_2_ planes. As shown in Fig. 3c, *δ*CDW_stripe_(*H*_⊥_) can account for the overall CDW enhancement at *H*_⊥_ ~ 10 T. Comparison to prior CDW measurements^22^ shows *δ*CDW_stripe_(*H*_⊥_) also nicely describes the CDW evolution at smaller magnetic fields *H*_⊥_ < 10 T (see Supplementary Fig. 11), suggesting that in the low field regime < ~ 10 T, the enhancement to the overall CDW is mainly due to CDW_stripe_, while the CDW_SRO_ intensity is relatively constant. Considering that CDW_SRO_ is expected to be strengthened as the magnetic field suppresses superconductivity^16^, the volume fraction for CDW_SRO_ has likely been reduced by the field. This is consistent with *μ*SR measurement that suggests the magnetic field increases the stripe order volume fraction^39^.

At larger field *H*_⊥_ > 10 T, we observe a sudden increase in the overall CDW intensity that cannot be accounted for by *δ*C*DW*_stripe_ (Fig. 3c). We conclude this originates from CDW_SRO_. The field range where this enhancement occurs coincides with the vortex-melting field *H*_*m*_ ~ 10 T inferred in transport measurements^40,41^. Superconducting vortices which were pinned in the vortex solid at lower magnetic fields become mobile above *H*_*m*_. This results in the loss of superconductivity due to the destruction of long-range phase coherence^40^. Considering the interplay between CDW_SRO_ and superconductivity (Fig. 2), the sudden increase of the CDW amplitude *δ*CDW_SRO_ above *H*_*m*_ (or, equivalently, a suppression of CDW_SRO_ upon entering the vortex-solid phase) implies a strong response of the CDW to the state of the vortices, whether pinned or mobile. Our result is complementary to recent theoretical and experimental studies that reveal an intricate interaction between superconductivity and topological defects in the coexisting CDW order^42–44^. We show that mobile topological defects in the superconducting order, the vortices, greatly enhances the CDW. It will be interesting to measure LSCO samples of different doping levels in the future to investigate how the detailed structure of the vortex solid affects the response of the CDW order at the vortex-melting transition^45^.

## Discussion

Both sets of our x-ray measurements (the *T*-dependence in zero-field, and the *H*-dependence at low-*T*) reveal the presence of two regions: regions that favor static spin-charge stripe order and regions that favor superconductivity (where the latter also harbor short-range CDW_SRO_). The stripe phase competes with uniform superconductivity, consistent with model calculations which reveal a near degeneracy between the superconducting and the stripe states^46^. Interestingly, a small change in the Hamiltonian can drive the system between these two distinct phases through an intermediate state featuring phase separation^47^. In LSCO, the presence of dopant disorder or structural inhomogeneity may play a role in stabilizing both phases simultaneously within the same sample.

The observed behavior of the CDW_SRO_ within the majority superconducting regions is particularly interesting. While CDW peaks have been observed in the normal and superconducting states of other cuprates, here, we find evidence that enhanced CDW_SRO_ is linked to the high-field vortex-liquid state. Noting that similar CDW peaks are observed in zero field in a broad temperature range above *T*_*c*_, one finds an interesting connection with Nernst effect measurements. Despite different interpretations regarding the origin of the Nernst signal at higher temperatures, there is consensus that for a temperature range ~30 K above *T*_*c*_ in LSCO the Nernst signal can be attributed to superconducting fluctuations^40,48,49^. This is the same temperature range where the CDW becomes significant (Fig. 1c). By plotting the CDW intensity in the vortex-liquid state (in a field of 24 T) in Fig. 1c, we find good agreement with an extrapolation of the zero-field intensity from above *T*_*c*_ to low temperatures. This connection between the CDW in the vortex liquid to that above *T*_*c*_ is consistent with the interpretation that the vortex-liquid state is continuously connected to the non-superconducting state above *T*_*c*_, where both regions of the *H* − *T* phase diagram possess a large Nernst signal due to mobile vortices^40^. Our results indicate that mobile vortices and CDW_SRO_ correlations are both inherent to the state where superconducting long-range phase coherence is lost. Within a phase-disordering scenario for the loss of superconductivity^40^, short-range two-dimensional CDW correlations appear to be compatible with local superconducting pairing.

These results reveal a distinction between the two types of charge order in the cuprates: CDW_stripe_ and CDW_SRO_. CDW_stripe_ and the associated static stripe state is most prominent in the La_2_CuO_4_-based cuprates, and is clearly competitive with uniform superconductivity. The ubiquitous CDW_SRO_, on the other hand, coexists with local superconductivity and may even aid the formation of vortices^50,51^. It is long range superconducting phase coherence that simultaneously suppresses CDW_SRO_ and the presence of mobile vortices. The apparent sensitivity of CDW correlations to superconducting phase coherence further suggests a unified quantum description of the density waves and superconductivity in cuprate superconductors^2,8,24^.

## Methods

### Sample preparation

High-quality single crystalline La_1.885_Sr_0.115_CuO_4_ samples were grown by the traveling solvent floating zone method. The typical growth rate was 1.0 mm h^−1^ and a 50–60 mm-long crystal rod was successfully obtained. A 10 mm-long crystalline piece from the end part of the grown rod was annealed in oxygen gas flow to minimize oxygen deficiencies. The superconducting transition temperature *T*_*c*_ of the sample is determined to be 27.5(2) K (Supplementary Fig. 2). We focus on the CDW peak near (2.23,0,5.5) r.l.u. (reciprocal space is denoted using the tetragonal unit cell, a = b = 3.77 Å, c = 13.25 Å), where the CDW intensity is strong^20^ and the scattering geometry allows a large magnetic field projection ( ~ 82%) along the crystallographic *c*-axis (perpendicular to the CuO_2_ planes). The CDW peaks were initially confirmed by resonant soft x-ray scattering at SSRL^18^. A 1 × 0.5 × 0.5 mm^3^ sample was oriented using Laue x-ray diffraction and polished to the desired dimensions with [2.23,0,5.5] direction normal to the scattering surface. The vertical scattering plane is spanned by the nominal [2.23,0,5.5] and [0,1,0] directions. In this geometry, the magnetic field direction is ~ 35. 2^∘^ tilted away from the crystalline *c*-axis.

### XFEL measurement

The x-ray scattering experiment was carried out on the X-ray Correlation Spectroscopy (XCS) instrument at the Linac Coherent Light Source (LCLS) at the SLAC National Accelerator Laboratory (Supplementary Fig. 1). Horizontally polarized x-ray beam with incident energy of 8.8 keV was used with the pink beam set up. The photon energy was chosen to be just below the Cu *K*-edge to reduce fluorescence background. A split coil pulsed magnet was used to provide large magnetic fields. Femtosecond x-ray pulses were synchronized with the magnetic field pulse (~1 ms duration) such that one photon pulse arrives on the sample at the maximum magnetic field strength. The orientation of the sample was determined by measuring the (204) and (206) nuclear Bragg peaks, which was then used to convert the pixel coordinate to the reciprocal-space coordinate (Supplementary Fig. 3). The CDW temperature dependence was probed upon warming. The magnetic-field-dependent measurements were carried out at the lowest temperature achievable of 6.5 K, where the largest field-induced effect is expected. The sample rotation was fixed at the CDW rocking scan peak center (Supplementary Fig. 4). For each magnetic-field run, 10 measurements at zero field were taken immediately before and after the field pulse to provide an accurate zero-field reference (Supplementary Fig. 1). While for zero-field measurements data can be taken continuously at 120 Hz x-ray pulse frequency, measurements in magnetic fields require extra time to cool down the magnet after each magnetic-field pulse. For example, at 24 T the cool-down time was typically ~15 minutes between magnetic pulses. This imposed a constraint on the statistical quality of the high-field data achievable during the finite beam time.

## Supplementary information

Supplementary Information

Peer Review File

## Data Availability

All data needed to evaluate the findings in this paper are present in the paper and/or the Supplementary Information. Further data sets are available from the corresponding authors upon reasonable request.
