# Supplementary material for: Enhanced charge density wave with mobile superconducting vortices in La1.885Sr0.115CuO4
Source: Nat Commun. 2023 Feb 9;14:733. doi: 10.1038/s41467-023-36203-x (PMC9911724; doi:10.1038/s41467-023-36203-x)
Supplement: Supplementary file 1 — Supplementary Information [file 41467_2023_36203_MOESM1_ESM.pdf]

# Enhanced charge density wave with mobile superconducting vortices in $La_{1.885}Sr_{0.115}CuO_4$ - Supplementary Information


J.-J. Wen,[1] W. He,[1,2] H. Jang,[3,4] H. Nojiri,[5] S. Matsuzawa,[5] S. Song,[6] M. Chollet,[6] D. Zhu,[6] Y.-J. Liu,[3] M. Fujita,[5] J. M. Jiang,[1,7] C. R. Rotundu,[1] C.-C. Kao,[8] H.-C. Jiang,[1] J.-S. Lee,[3] and Y. S. Lee[1,7]

[1]*Stanford Institute for Materials and Energy Sciences, SLAC National Accelerator Laboratory, 2575 Sand Hill Road, Menlo Park, CA 94025, USA*

[2]*Department of Materials Science and Engineering, Stanford University, Stanford, CA 94305, USA*

[3]*Stanford Synchrotron Radiation Lightsource, SLAC National Accelerator Laboratory, Menlo Park, CA 94025, USA*

[4]*PAL-XFEL, Pohang Accelerator Laboratory, Gyeongbuk 37673, South Korea*

[5]*Institute for Materials Research, Tohoku University, Katahira 2-1-1, Sendai, 980-8577, Japan*

[6]*Linac Coherent Light Source, SLAC National Accelerator Laboratory, Menlo Park, CA 94025, USA*

[7]*Department of Applied Physics, Stanford University, Stanford, CA 94305, USA*

[8]*SLAC National Accelerator Laboratory, Menlo Park, CA 94025, USA*

**CONTENTS**


I. Experimental Setup 2

II. Sample Characterization 3

III. Nuclear Bragg Peaks and Structural Twin Domains 4

IV. Additional Characterizations of CDW Spatial Correlations 6

V. CDW Temperature Dependence for LSCO in Prior Studies 7

VI. Comparison of Fitting Quality between Single-peak and Two-component Fittings 8

VII. Comparison to CDW in $La_{1.875}Ba_{0.125}CuO_4$ 9

VIII. Evidence for Proportionality between $CDW_{stripe}$ and SDW in Magnetic Fields 10

IX. Additional Characterizations of CDW Field Dependence 11

X. Comparison to CDW Field Dependence in YBCO 12

References 17


## I. EXPERIMENTAL SETUP

The x-ray scattering experiment was carried out on the X-ray Correlation Spectroscopy (XCS) instrument at the Linac Coherent Light Source (LCLS) at the SLAC National Accelerator Laboratory (Supplementary Fig. 1a). During each high-field measurement, 10 zero-field measurements were taken before and after the magnetic field pulse respectively (a total of 20 zero-field measurements), which provide the corresponding zero-field reference. A schematic of the measurement sequence is shown in Supplementary Fig. 1b. Scattered photons were detected with the CSPAD area detectors. To account for the photon flux variations between x-ray pulses, each scattering pattern was normalized such that the total photon count in the rectangular area indicated as background in Supplementary Fig. 1a, where the intensities are mostly due to fluorescence, is $10^4$ per x-ray pulse.

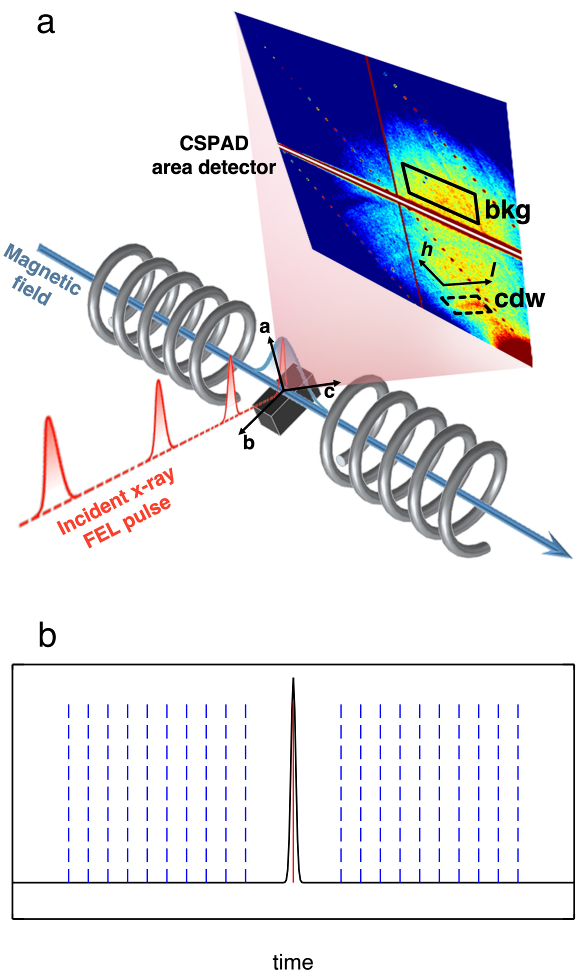


Supplementary Figure 1. (a) A schematic of the experimental setup. In the raw scattering pattern, the dashed box indicates the CDW signal, and the solid box shows the background region used for photon flux normalization. The intensities near the lower right corner are due to tail of the (206) Bragg peak. The diagonal dots are features of the detector. (b) High-field measurement schematic. The black solid peak represents the magnetic-field pulse. Dashed blue vertical lines show the 20 zero-field measurements taken before and after the field pulse, and the red solid vertical line represents the x-ray pulse arrives at the peak of the magnetic field.

## II. SAMPLE CHARACTERIZATION

The superconducting transition temperature ($T_c$) of the $La_{1.885}Sr_{0.115}CuO_4$ (LSCO) sample was determined by magnetic susceptibility using a Physical Properties Measurement System from Quantum Design, Inc. The real part of the magnetic susceptibility, $\chi'$, measured with 4 Oe AC magnetic field at 1000 Hz, is shown in Supplementary Fig. 2. $T_c$ as

defined by the midpoint of the transition is found to be 27.5(2) K, consistent with previous measurements on samples with similar doping levels[1].

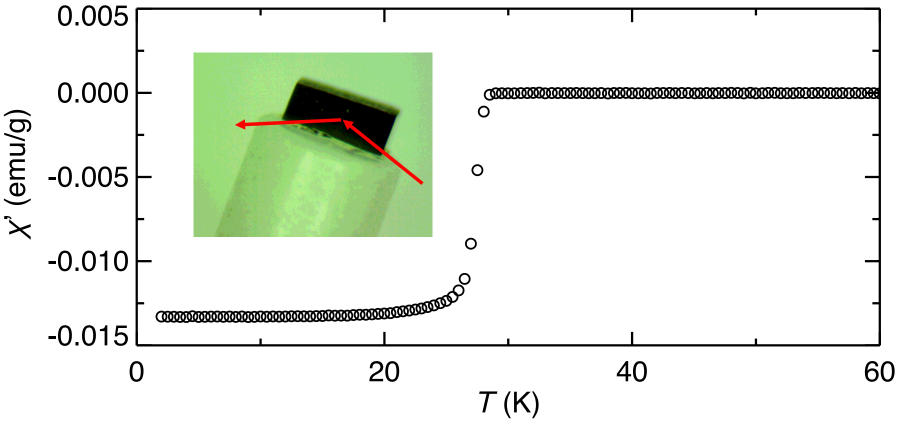


Supplementary Figure 2. Magnetic susceptibility of the LSCO sample, showing a sharp superconducting transition at $T_c = 27.5(2)$ K. Inset shows a pictures of the sample ($1 \times 0.5 \times 0.5$ mm$^3$) mounted on a sapphire rod. The red arrows indicate how x-ray beam scatters off the polished sample surface.

## III. NUCLEAR BRAGG PEAKS AND STRUCTURAL TWIN DOMAINS

The orientation of the sample was determined by measuring the (204) and (206) nuclear Bragg peaks, which is then used to convert the data from the detector pixel coordinate to the reciprocal space coordinate of the sample. Supplementary Figs. 3a-c show the $h-$, $k-$, and $l-$ cuts through the (206) Bragg peak measured at 28 K. The peak widths as determined by fitting to these cuts are used to estimate the instrumental resolution at the nearby (2.235,0,5.5) CDW peak position.

LSCO sample generally develops four structural twin domains below the tetragonal-orthorhombic structural transition temperature ($\sim 250$ K for the doping level studied here[1]), which can be considered as two pairs of twin domains that are related by 90° rotation[2]. As a result, we expect to see three (206) peaks that are split along the $k$-direction: a central peak due to one pair of domains, and two side peaks due to the other pair. As shown in Supplementary Fig. 3b, the two side peaks are much weaker compared to the central peak ($\sim$7 % as estimated by ratio of the integrated peak intensities), suggesting that our LSCO sample is dominated by just one pair of twin domains, similar to the situation observed in

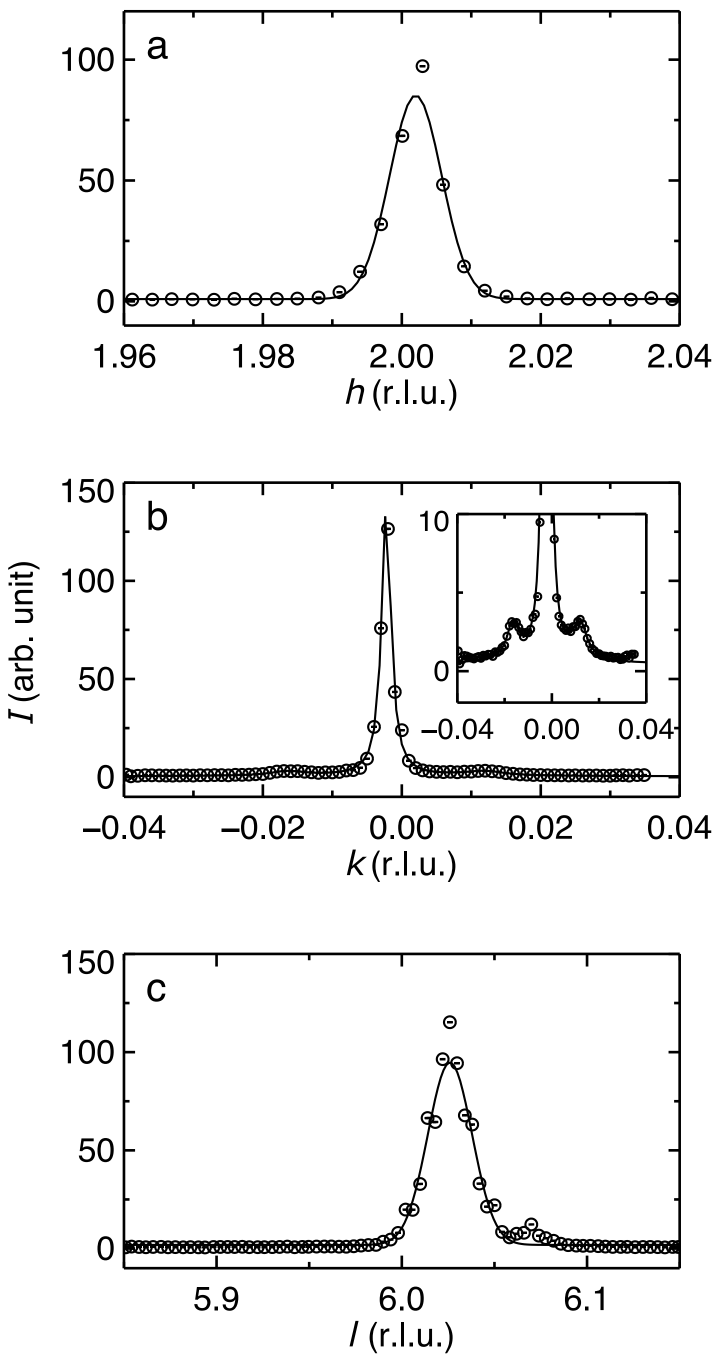


Supplementary Figure 3. (a-c) Cuts through the (206) nuclear Bragg peak along $h-$, $k-$, and $l-$ directions, respectively. Inset to (b) is a zoom-in plot of the $k-$cut, showing that in addition to a main central peak due to the dominant pair of structural domains, there are two weak side peaks due to the minor domain pair. Solid lines are fits to the data. Error bars represent one standard deviation.

a previous study[2].

## IV. ADDITIONAL CHARACTERIZATIONS OF CDW SPATIAL CORRELATIONS

The total scattering intensity of the CDW peak, defined as the sum of the scattering intensities within the dashed rectangle indicated in Supplementary Fig. 1a, measured as a function of the sample rotation angle ($\theta$) at 28 K is shown in Supplementary Fig. 4. For high-field measurements the sample rotation angle is fixed at the peak center of $\theta = 30.8°$.

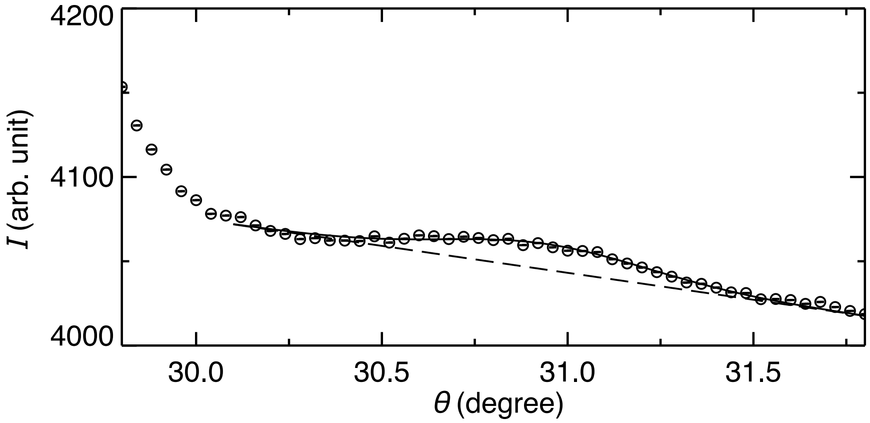


Supplementary Figure 4. $\theta$-scan around the CDW position measured at 28 K. The solid line is a fit to the data, and the dashed line indicates the linear background obtained from the fitting. Error bars represent one standard deviation.

We can take a closer look at the CDW spatial correlations by projecting the CDW $\theta$-scan data onto reciprocal space coordinate. As shown in Fig. 1a of the main text, the data projected onto $hl$ plane shows a rod of scattering intensities along the $l$ direction. Cutting the CDW peak at different $l$ and plotting the $l-$dependent CDW intensities further corroborate the weak $l$ dependence, as shown in Supplementary Fig. 5. Applying magnetic field up to 24 T does not induce any significant change to the inter-$CuO_2$ plane CDW correlations, as shown in the almost field-independent $l$ dependence of the CDW intensities (Supplementary Fig. 5b).

Integrating the CDW intensities along $l$ and projecting the data onto $hk$ plane reveal the CDW correlations within the $CuO_2$ plane, as shown in Supplementary Fig. 6. Two CDW peaks centered at $h$ of $q_{\rm cdw} = 0.236(2)$ r.l.u. but split along the $k-$direction, are clearly observed. They can be associated with the two orthorhombic structural domains (the dominant domain pair) discussed in Supplementary Fig. 3. The size of the splitting, $\theta_Y \sim$

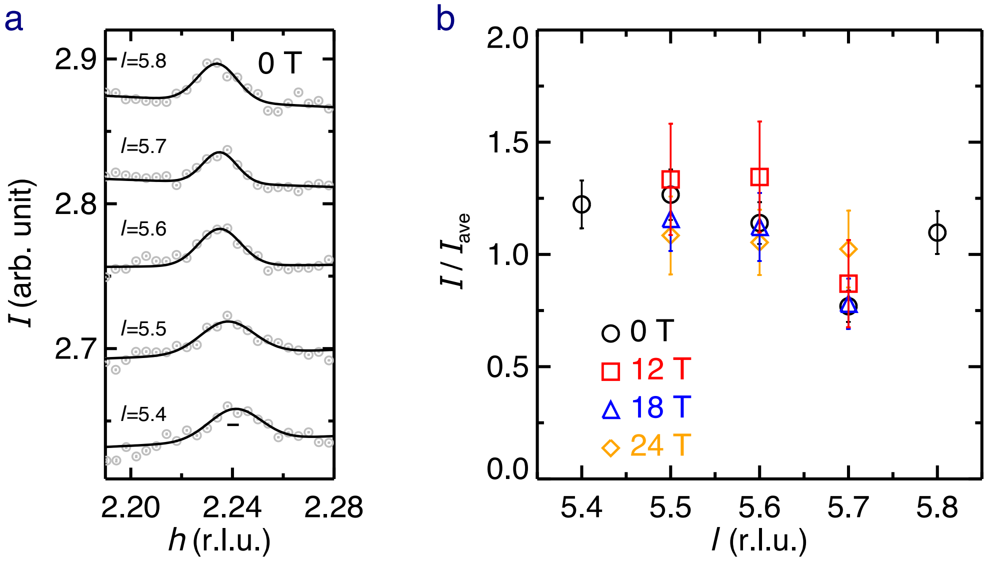


Supplementary Figure 5. (a) $h-$cuts at different $l$ positions of the CDW peak measured at 0 T, 6.5 K. Solid lines are fits to the data. (b) $l-$dependent CDW intensities measured at various magnetic fields, 6.5 K. To account for overall increase in CDW intensities at high fields, intensities at each field are normalized to the respective CDW intensity averaged over $l$ between 5.5 and 5.8 r.l.u.. Error bars represent one standard deviation.

$\delta_k/2q_{\rm cdw} = 2.5(1)^\circ$ at 6.5 K, however, is an order of magnitude larger than what one expects from simple structural twinning effect[3], and suggests that the CDW does not propagate along the Cu-Cu bond direction, consistent with previous soft x-ray scattering measurements[3,4]. $\theta_Y$ of $2.5(1)^\circ$ is similar to that for the SDW order ($\sim 3^\circ$) in $La_{1.88}Sr_{0.12}CuO_4$[2], providing further support for the inter-locking between CDW and SDW orders as in the spin-charge stripe order[5,6]. The splitting persists at high temperatures in the absence of the SDW order [$\theta_Y = 2.1(1)^\circ$ at 28 K, see Supplementary Fig. 6b]. This suggests a common origin for $\theta_Y$ in $\rm CDW_{SRO}$ and $\rm CDW_{stripe}$, such as anisotropy in the second-neighbor electron hopping in the underlying Hamiltonian[7].

## V. CDW TEMPERATURE DEPENDENCE FOR LSCO IN PRIOR STUDIES

In Supplementary Fig. 7 we summarize the temperature dependence of CDW intensity and peak width for $La_{1.88}Sr_{0.12}CuO_4$ reported in prior studies[3,8,9]. Consistent with our data, these data all exhibit contradictory behavior between the CDW intensity and the peak

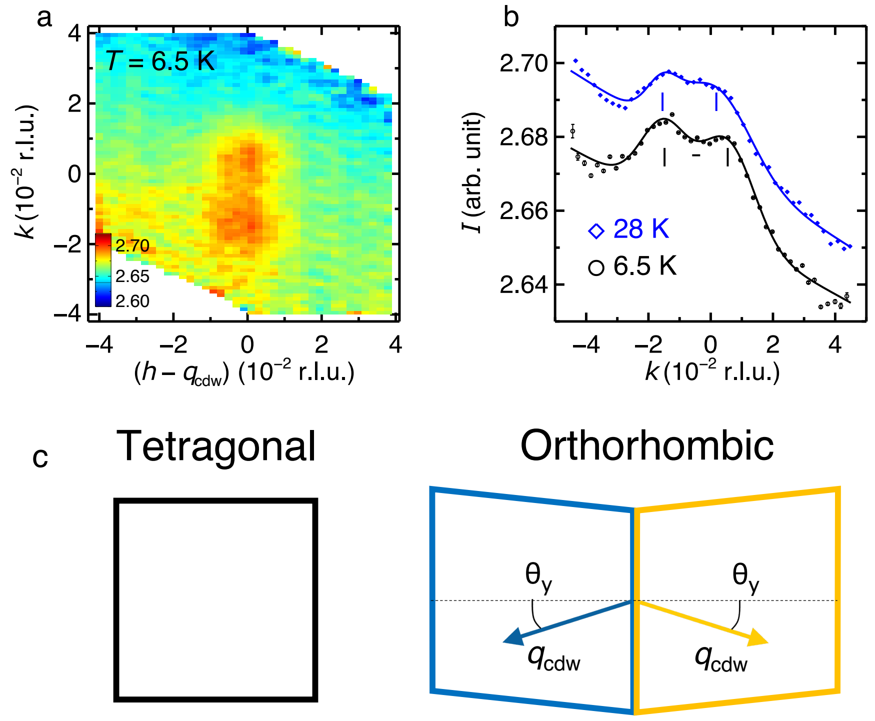


Supplementary Figure 6. (a) CDW intensities projected onto $hk$ plane, measured at 0 T, 6.5 K. (b) $k-$cuts of the CDW intensities measured at 6.5 K and 28 K. Solid lines are fits to the data. (c) Schematic illustration of how two CDW peaks arise from two orthorhombic structural domains. Error bars represent one standard deviation.

width. The intensity depresses/saturates below $T_c$, while the peak width keeps decreasing monotonically.

## VI. COMPARISON OF FITTING QUALITY BETWEEN SINGLE-PEAK AND TWO-COMPONENT FITTINGS

We can judge the goodness of fit for the single-peak and two-component fittings by comparing reduced $\chi^2$. As shown in Supplementary Fig. 8, the two-component fittings are consistently better than the single-peak fittings. Note that after fixing the peak width for $\mathrm{CDW_{stripe}}$ and $\mathrm{CDW_{SRO}}$, and using a common peak center for these two components, the number of fitting parameters is the same in the two-component fitting as that in the single-peak fitting.

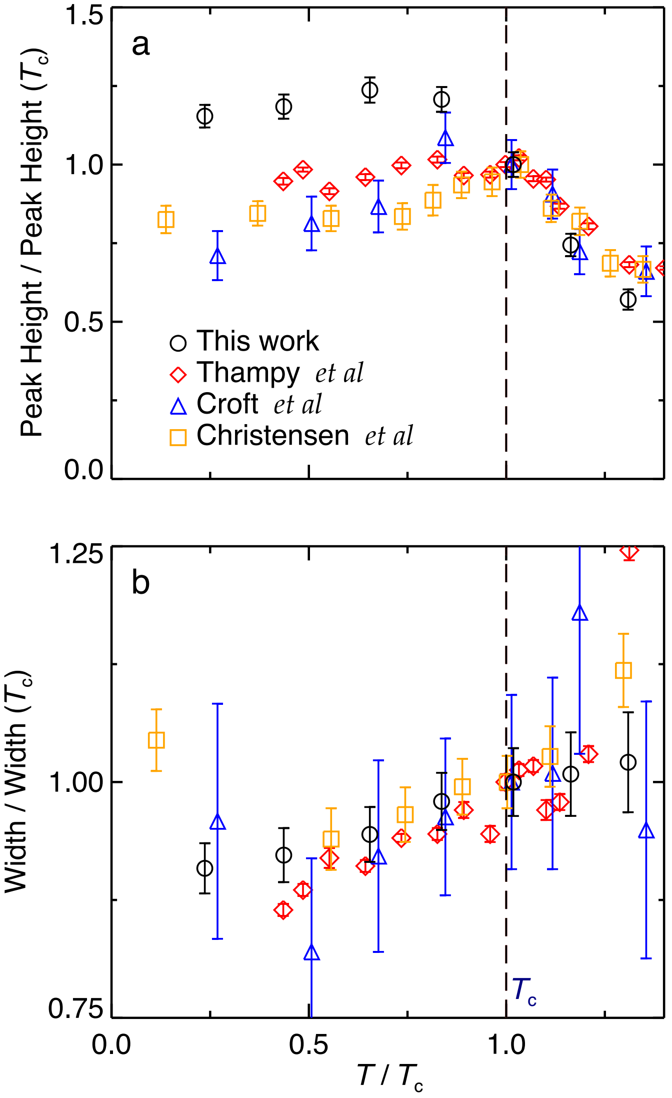


Supplementary Figure 7. Temperature dependence of (a) CDW intensity and (b) CDW peak width for $La_{1.88}Sr_{0.12}CuO_4$[3,8,9]. Data from different studies are normalized by the value $\sim T_c$, respectively. Error bars represent one standard deviation.

## VII. COMPARISON TO CDW IN $LA_{1.875}BA_{0.125}CUO_4$

Assuming the volume fraction for the stripe-order-dominant regions in LSCO in the low-temperature limit is 18% as deduced from $\mu$SR measurements[10], we can estimate the $CDW_{stripe}$ intensity per unit volume in the stripe ordered regions (up to an overall scale factor) as $I_{stripe}(T = 6.5\text{ K})/0.18 = 8(3) \times 10^{-4}$. Similarly, the $CDW_{SRO}$ intensity in the superconductivity-dominant regions is $I_{SRO}(T = 6.5\text{ K})/0.82 = 5.7(8) \times 10^{-4}$. Here $I_{stripe}$ and $I_{SRO}$ are the intensities extracted in the two-component analysis. Their ratio of 1.4(6)

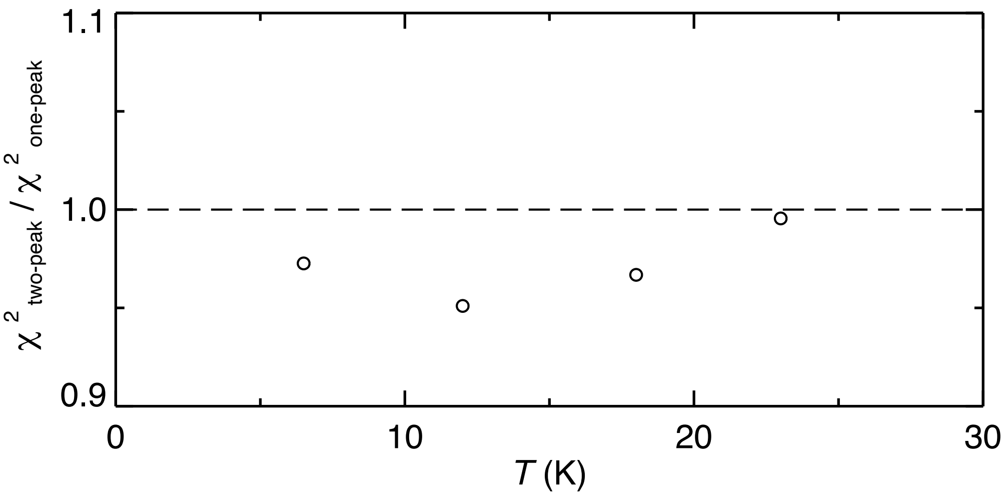


Supplementary Figure 8. Comparison of reduced $\chi^2$ for single-peak and two-component fittings.

is consistent with our starting assumption that the CDW is enhanced in the stripe ordered regions while suppressed in the superconductivity-dominant regions.

It is also interesting to compare the CDW intensities in $La_{1.885}Sr_{0.115}CuO_4$ to that in $La_{1.875}Ba_{0.125}CuO_4$ (LBCO), a prototypical stripe ordered cuprate where the stripe order is expected to be strongest and the magnetic volume is estimated to be $\sim 100\%$[10]. In a previous study it was found that the CDW intensity in LSCO is $\sim 1/4$ of that in LBCO[3]. Focusing on the stripe ordered regions, the unit volume $\text{CDW}_{\text{stripe}}$ intensity in LSCO compared to that in LBCO is $\sim \{1/[0.18 + (0.82/1.4)]\}/4 \sim 0.3$, consistent with a stronger stripe order in LBCO.

## VIII. EVIDENCE FOR PROPORTIONALITY BETWEEN $\text{CDW}_{\text{stripe}}$ AND SDW IN MAGNETIC FIELDS

Here we elaborate on the main assumption we made to analyze the magnetic field dependence of the CDW in $La_{1.885}Sr_{0.115}CuO_4$, that $\text{CDW}_{\text{stripe}}(H) \sim \text{SDW}(H)$. First, this is consistent with our finding from the two-component analysis that $\text{CDW}_{\text{stripe}}(T) \sim \text{SDW}(T)$ in zero magnetic field (Fig.2a of the main text). Second, in $La_{1.88}Sr_{0.12}CuO_4$, it is found that at $\sim 7$ T, the field-enhanced CDW intensities, which in our model is mostly due to $\text{CDW}_{\text{stripe}}$, is proportional to the field-enhanced SDW intensities, as shown in Fig. 2d in ref. 9. Third, in canonical stripe ordered cuprate material $La_{2-x}Ba_xCuO_4$, it is found that for $x = 1/8$, where the stripe order is strongest, magnetic field has negligible enhancement

effect on neither CDW nor SDW[11]. For $La_{1.905}Ba_{0.095}CuO_4$, where clear field enhancement to the stripe order has been observed, CDW and SDW are enhanced by the same proportion, i.e. $CDW_{stripe}(H)/CDW_{stripe}(0) = SDW(H)/SDW(0)$, as shown in Supplementary Fig. 9, using data from refs. 11-12.

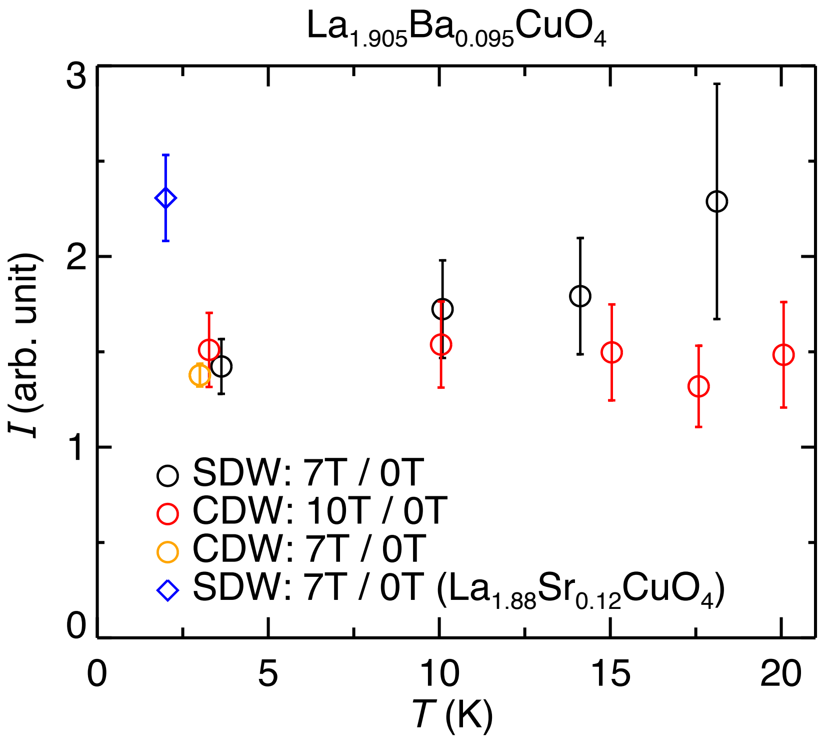


Supplementary Figure 9. Proportionality between magnetic-field-enhanced CDW and SDW for $La_{1.905}Ba_{0.095}CuO_4$[11,12]. For comparison, we also plot the field enhancement of SDW in $La_{1.88}Sr_{0.12}CuO_4$ at similar magnetic field[13]. Error bars represent one standard deviation.

## IX. ADDITIONAL CHARACTERIZATIONS OF CDW FIELD DEPENDENCE

As described in Section I. Experimental Setup, for each pulsed field measurement the corresponding zero-field data have 20 times more counting time compared to the data collected in field. To take advantage of the higher statistics zero-field data, we fitted the in-field data and the corresponding zero-field data simultaneously, with the constraint that both have the same linear background under the reasonable assumption that the background does not vary with applied magnetic fields, as shown in Supplementary Fig. 10.

In Supplementary Fig. 11 we compare the existing data of field-dependent CDW in

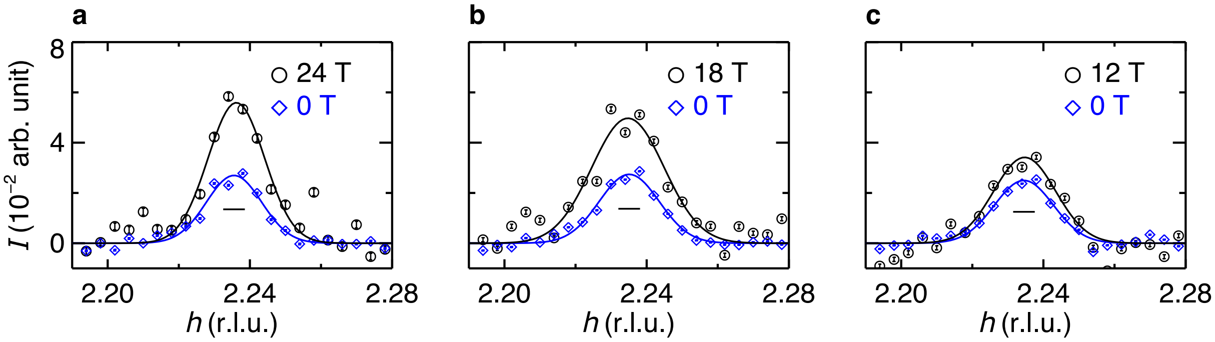


Supplementary Figure 10. Field-dependent CDW peaks and the corresponding zero-field CDW peaks. Solid lines are fits to the data, as described in the text. A linear background has been subtracted. Error bars represent one standard deviation.

$La_{1.88}Sr_{0.12}CuO_4$ (up to 10 T)[9] with our measurements. As shown in Supplementary Fig. 11a, the prior data is also well described by $CDW_{stripe}$, providing additional support for our proposition that in the low field regime, the enhancement to the CDW mostly comes from $CDW_{stripe}$, while $CDW_{SRO}$ remains nearly unchanged. Assuming that $CDW_{SRO}$ follows a step-like enhancement at $H_m$, and that the peak widths for $CDW_{SRO}$ and $CDW_{stripe}$ remain the same as their zero-field values, we can simulate the field dependence of both intensity and width. As shown in Supplementary Fig. 11, such a simple model provides a semi-quantitative description of the prior data and our data.

## X. COMPARISON TO CDW FIELD DEPENDENCE IN YBCO

$CDW_{SRO}$ in LSCO is similar to the short-range CDW ($\sim$ half-integer $l$) observed in YBCO in that both are suppressed below the superconducting transition $T_c$[14]. In YBCO it is found that the CDW intensities increase linearly with applied magnetic fields in the low-field regime[15]. If there is no spin-charge stripe order, we would expect $CDW_{SRO}$ in LSCO to increase with applied field even at low fields, like YBCO. However, the stripe order in LSCO is also enhanced by applied magnetic fields, and according to $\mu$SR measurements this enhancement is mostly due to increase in volume fraction of the stripe ordered regions[16]. Correspondingly, the volume fraction of the $CDW_{SRO}$ region is reduced. Therefore, the magnetic-field-induced enhancement to $CDW_{SRO}$, due to weakening of superconductivity, is offset by the reduction in the volume fraction. Our data and analysis suggests that the

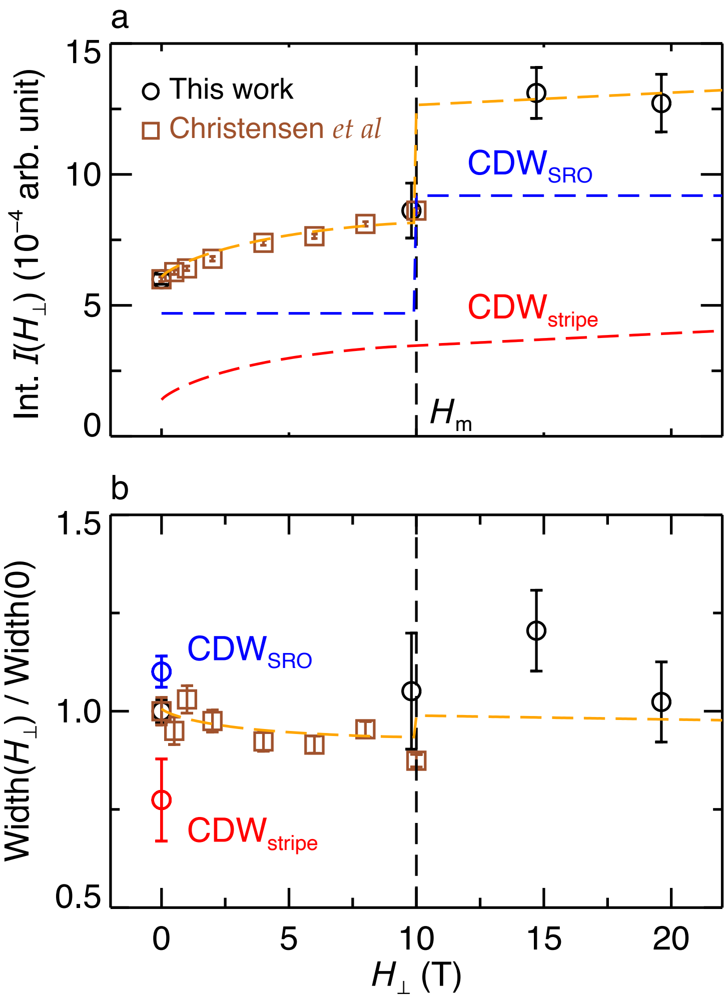


Supplementary Figure 11. (a) Magnetic-field dependence of the CDW intensities for LSCO. Brown square is the data reported in Christensen *et al*[9], shifted and scaled to match common data points at 0 T and ∼ 10 T of this work. Red dashed line is the $CDW_{stripe}$ inferred from SDW field dependence, as described in the main text. Blue dashed line is a simple description of the $CDW_{SRO}$, assuming a step-like enhancement at $H_m$. Orange dashed line is the total CDW intensity (sum of $CDW_{stripe}$ and $CDW_{SRO}$). (b) Magnetic-field dependence of the CDW peak width for LSCO. Brown square is the data reported in Christensen *et al*[9]. Orange dashed line is the peak width extracted by fitting to the simulated total CDW peak, as described in the text. Blue and red circles are the peak widths for $CDW_{SRO}$ and $CDW_{stripe}$ in zero magnetic field, as extracted in the two-component analysis in the main text. Error bars represent one standard deviation.

net effect is a weak field dependence of $CDW_{SRO}$ intensity in the low-field regime. As such, the $CDW_{SRO}$ behavior in LSCO may be seen as consistent with the short-range CDW in

YBCO, and it is the rapid reduction in $\mathrm{CDW_{SRO}}$ volume fraction in the low-field regime that renders a weak field dependence for $\mathrm{CDW_{SRO}}$ in LSCO.

At larger fields, we find a large enhancement to the $\mathrm{CDW_{SRO}}$ intensities upon entering the vortex-liquid state in LSCO. A close inspection of the field dependence of the short-range CDW in YBCO (reproduced in Supplementary Figs. 12a-b using data in ref. 18) across the corresponding vortex-melting field ($H_\mathrm{m}$) also suggests a CDW anomaly around $H_m$. As shown in Supplementary Fig. 12a, the linear CDW intensity enhancement at low fields turns into a plateau above $H_m$, a field that is only $\sim 60\%$ of $H_{c2}$[17]. The CDW correlation length shown in Supplementary Fig. 12b is also consistent with an anomaly $\sim H_\mathrm{m}$. Together with our data, this suggests that a strong response of short-range CDW to the vortex-melting transition could be a common phenomenon in cuprate superconductors.

It is found in YBCO superconductors that high magnetic field induces a three-dimensional CDW peak at integer $l$ positions[18,23,24]. As shown in Fig. 3a of the main text, there is no apparent magnetic-field-enhanced CDW intensities at $l = 6$ at 24 T in LSCO. Systematic $h-$cuts at $l = 6$ at various fields, as shown in Supplementary Fig. 13, indicate the absence of CDW intensity at $l = 6$ up to at least 24 T.

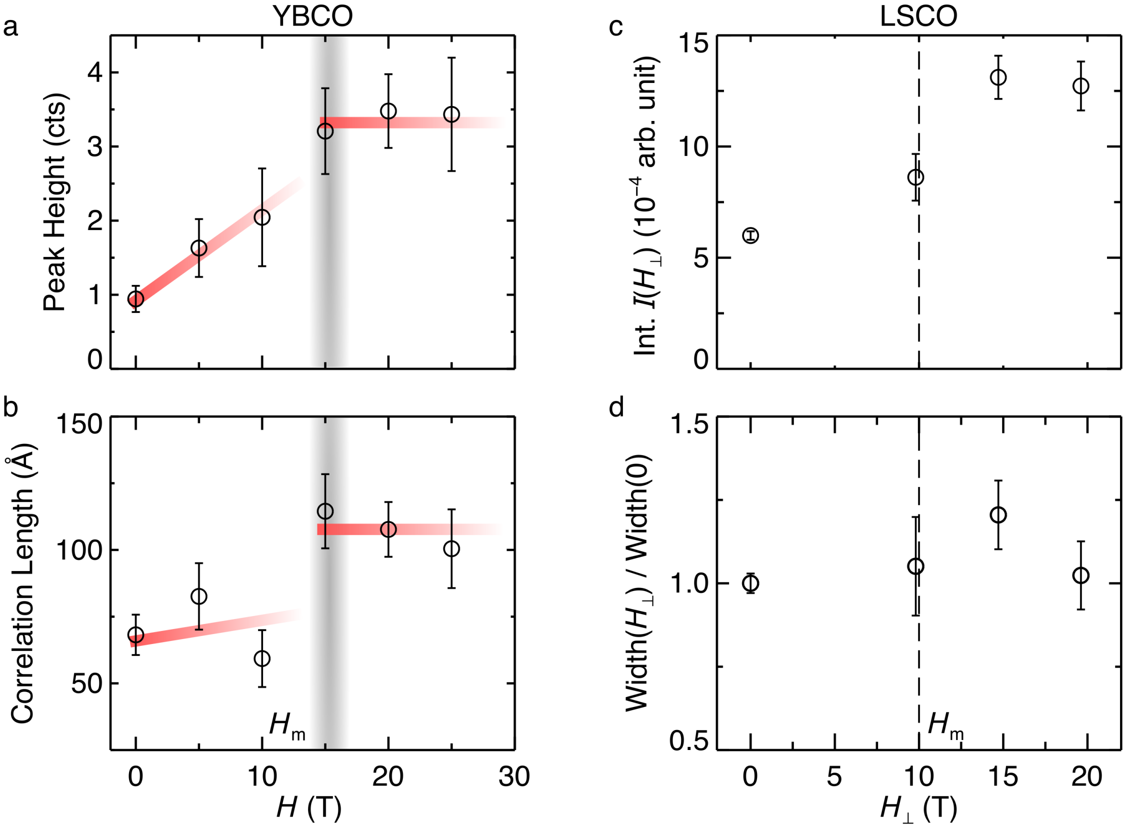


Supplementary Figure 12. Magnetic-field dependence of short-range CDW ($l \sim 0.5$ r.l.u.) in $YBa_2Cu_3O_{6.67}$[18] (a,b) compared to data for $La_{1.885}Sr_{0.115}CuO_4$ in this work (c,d). Vertical lines are the corresponding vortex-melting field $H_m$[19–22]. A range of $H_m$ for $YBa_2Cu_3O_{6.67}$ at $\sim 10$ K has been reported using various experimental techniques and different ways of determining $H_m$, as illustrated by the thick vertical line in (a,b)[19–21]. Considering that the vortex-melting transition typically has width of a few Tesla[19–21], the reported $H_m$ for YBCO is consistent with our association of the CDW anomaly with the vortex-melting transition. Red lines in (a,b) are guides to the eye. Error bars represent one standard deviation.

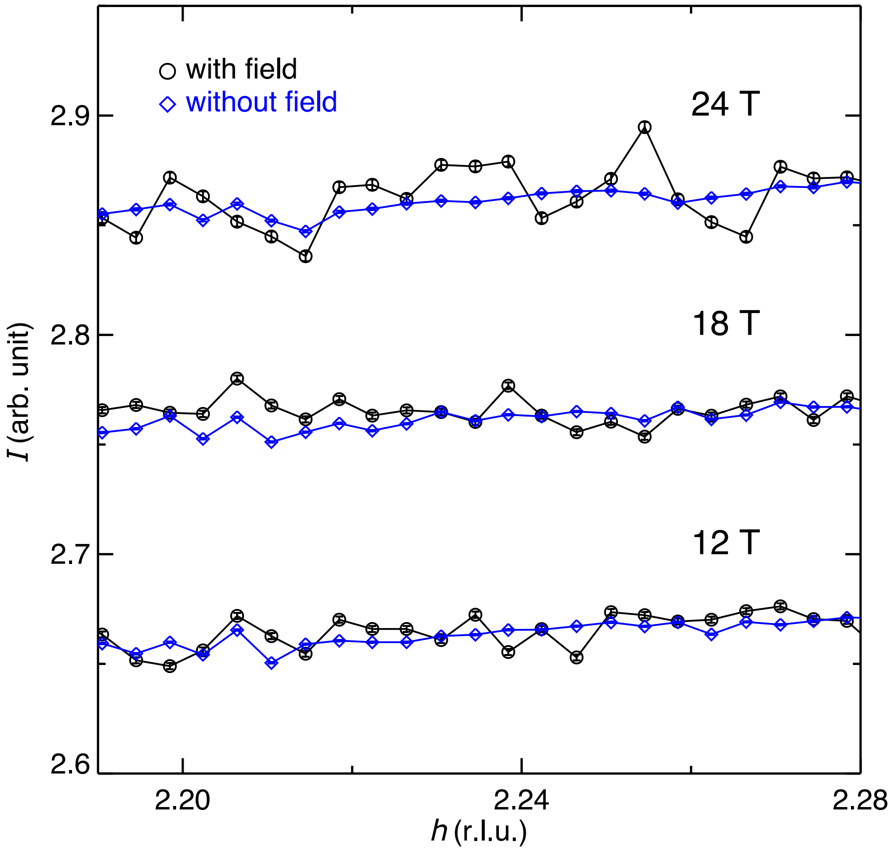


Supplementary Figure 13. $h-$cuts at $l = 6$ at various magnetic fields and corresponding measurements at zero magnetic field. Data for different fields are shifted for clarity. Error bars represent one standard deviation.

[1] Yamada, K. *et al.* Doping dependence of the spatially modulated dynamical spin correlations and the superconducting-transition temperature in $La_{2-x}Sr_xCuO_4$. *Phys. Rev. B* **57**, 6165–6172 (1998).

[2] Kimura, H. *et al.* Incommensurate geometry of the elastic magnetic peaks in superconducting $La_{1.88}Sr_{0.12}CuO_4$. *Phys. Rev. B* **61**, 14366–14369 (2000).

[3] Thampy, V. *et al.* Rotated stripe order and its competition with superconductivity in $La_{1.88}Sr_{0.12}CuO_4$. *Phys. Rev. B* **90**, 100510(R) (2014).

[4] Wen, J.-J. *et al.* Observation of two types of charge-density-wave orders in superconducting $La_{2-x}Sr_xCuO_4$. *Nat. Commun.* **10**, 3269 (2019).

[5] Tranquada, J. M., Sternlieb, B. J., Axe, J. D., Nakamura, Y. & Uchida, S. Evidence for stripe correlations of spins and holes in copper oxide superconductors. *Nature* **375**, 561–563 (1995).

[6] Tranquada, J. M. Spins, stripes, and superconductivity in hole-doped cuprates. *AIP Conf. Proc.* **1550**, 114–187 (2013).

[7] He, W. *et al.* Prevalence of tilted stripes in $La_{1.88}Sr_{0.12}CuO_4$ and the importance of $t'$ in the Hamiltonian (2021). URL `https://arxiv.org/abs/2107.10264`.

[8] Croft, T. P., Lester, C., Senn, M. S., Bombardi, A. & Hayden, S. M. Charge density wave fluctuations in $La_{2-x}Sr_xCuO_4$ and their competition with superconductivity. *Phys. Rev. B* **89**, 224513 (2014).

[9] Christensen, N. B. *et al.* Bulk charge stripe order competing with superconductivity in $La_{2-x}Sr_xCuO_4$ (x=0.12) (2014). URL `https://arxiv.org/abs/1404.3192`.

[10] Savici, A. T. *et al.* Muon spin relaxation studies of incommensurate magnetism and superconductivity in stage-4 $La_2CuO_{4.11}$ and $La_{1.88}Sr_{0.12}CuO_4$. *Phys. Rev. B* **66**, 014524 (2002).

[11] Hücker, M. *et al.* Enhanced charge stripe order of superconducting $La_{2-x}Ba_xCuO_4$ in a magnetic field. *Phys. Rev. B* **87**, 014501 (2013).

[12] Wen, J. *et al.* Uniaxial linear resistivity of superconducting cycle $La_{1.905}Ba_{0.095}CuO_4$ induced by an external magnetic field. *Phys. Rev. B* **85**, 134513 (2012).

[13] Chang, J. *et al.* Tuning competing orders in $La_{2-x}Sr_xCuO_4$ cuprate superconductors by the application of an external magnetic field. *Phys. Rev. B* **78**, 104525 (2008).

[14] Ghiringhelli, G. *et al.* Long-range incommensurate charge fluctuations in (Y,Nd)$Ba_2Cu_3O_{6+x}$.

*Science* **337**, 821–825 (2012).

[15] Chang, J. *et al.* Direct observation of competition between superconductivity and charge density wave order in $YBa_2Cu_3O_{6.67}$. *Nat. Phys.* **8**, 871–876 (2012).

[16] Savici, A. T. *et al.* Muon spin relaxation studies of magnetic-field-induced effects in high-$T_c$ superconductors. *Phys. Rev. Lett.* **95**, 157001 (2005).

[17] Grissonnanche, G. *et al.* Direct measurement of the upper critical field in cuprate superconductors. *Nat. Commun.* **5**, 3280 (2014).

[18] Gerber, S. *et al.* Three-dimensional charge density wave order in $YBa_2Cu_3O_{6.67}$ at high magnetic fields. *Science* **350**, 949–952 (2015).

[19] LeBoeuf, D. *et al.* Lifshitz critical point in the cuprate superconductor $YBa_2Cu_3O_y$ from high-field hall effect measurements. *Phys. Rev. B* **83**, 054506 (2011).

[20] Ramshaw, B. J. *et al.* Vortex lattice melting and $H_{c2}$ in underdoped $YBa_2Cu_3O_y$. *Phys. Rev. B* **86**, 174501 (2012).

[21] Laliberté, F. *et al.* High field charge order across the phase diagram of $YBa_2Cu_3O_y$. *npj Quantum Mater.* **3**, 11 (2018).

[22] Frachet, M. *et al.* High magnetic field ultrasound study of spin freezing in $La_{1.88}Sr_{0.12}CuO_4$. *Phys. Rev. B* **103**, 115133 (2021).

[23] Jang, H. *et al.* Ideal charge-density-wave order in the high-field state of superconducting YBCO. *Proc. Nat. Acad. Sci. USA* **113**, 14645–14650 (2016).

[24] Chang, J. *et al.* Magnetic field controlled charge density wave coupling in underdoped $YBa_2Cu_3O_{6+x}$. *Nat. Commun.* **7**, 11494 (2016).
